# Cardiac troponin as a prognosticator of mortality in patients with sepsis: A systematic review and meta‐analysis

**DOI:** 10.1002/iid3.1014

**Published:** 2023-09-22

**Authors:** Peiqiu Zheng, Xing Wang, Tao Guo, Wei Gao, Qiang Huang, Jie Yang, Hui Gao, Qian Liu

**Affiliations:** ^1^ Department of Emergency Liyang Hospital of Chinese Medicine Changzhou Jiangsu China; ^2^ Department of Critical Care Medicine Affiliated Hospital of Nanjing University of Chinese Medicine Nanjing Jiangsu China; ^3^ Department of Emergency Affiliated Hospital of Nanjing University of Chinese Medicine Nanjing Jiangsu China; ^4^ Department of Critical Care Medicine Jiangsu Province Hospital on Integration of Chinese and Western Medicine Nanjing Jiangsu China; ^5^ Department of Critical Care Medicine Liyang Hospital of Chinese Medicin Changzhou Jiangsu China

**Keywords:** meta‐analysis, mortality, sepsis, troponin

## Abstract

**Background:**

The impact of cardiac troponin on the short‐term and long‐term prognosis of patients with sepsis remains uncertain. Therefore, we conducted a meta‐analysis to investigate the role of cardiac troponin as a potential indicator for sepsis mortality.

**Methods:**

We performed a comprehensive search for articles published before November 2022 using Google Scholar, PubMed, and Web of Science. Inclusion criteria for the studies were: (1) investigation of cardiac troponin, and (2) investigation of sepsis. Exclusion criteria included: (1) inability to obtain or calculate hazard ratio (HR) and 95% confidence interval (CI) for the relationship between cardiac troponin level and sepsis mortality, and (2) reviews, meta‐analyses, and case reports. Analysis of HRs and 95% CIs for the association between cardiac troponin level and sepsis mortality was conducted using STATA 12.0 software.

**Results:**

Our study included 24 prospective studies (comprising 20,457 sepsis patients) and 4 retrospective studies (comprising 1416 sepsis patients). Meta‐analysis demonstrated that elevated cardiac troponin levels were significantly associated with increased sepsis mortality using a random effects model (HR = 1.57, 95% CI 1.41−1.75). Moreover, elevated cardiac troponin levels were also significantly associated with increased hospital mortality of sepsis (HR = 1.35, 95% CI 1.19−1.53) and long‐term mortality of sepsis (HR = 1.96, 95% CI 1.51−2.55) using the random effects model.

**Conclusions:**

Overall, our finding revealed that elevated cardiac troponin for sepsis patients was a predictor of hospital and long‐term mortality. Clinicians may treat septic patients with elevated cardiac troponin more cautious to avoid extra death. Moreover, large clinical studies are warranted to validate this association.

## Graphical abstract

Overall, our finding revealed that elevated troponin for sepsis patients was a potential indicator of prognosis including hospital and long‐term mortality. Clinicians may treat septic patients with elevated troponin more cautious to avoid extra death. Moreover, large clinical studies are warranted to validate this association.

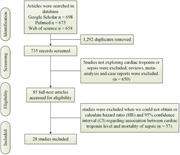

## 1 INTRODUCTION

Sepsis is a common disease among hospitalized patients in the intensive care unit (ICU) globally that comes with high mortality and long‐term disability.^1^ Sepsis is defined as a life‐threating organ dysfunction caused by the dysfunctional immune response to infection that affects over 30 million people annually and act as a primary cause of death in critical patients.^2^ The average mortality rates of 30‐day and 90‐day sepsis were 24.4% and 38.5% in Europe, North America and Australia, respectively.^3^ In China, sepsis affected approximately one‐fifth of intensive care beds were occupied by septic patients with a 90‐day mortality rate of 35.5% in 2017.^4^ A multicenter study showed that sepsis affected one‐third of ICU patients with a mortality rate of 55.7% in Brazil.^5^ In low‐ and middle‐income countries, sepsis mortality and morbidity may increase because of inadequate medical supplies, manpower and diagnostic capacity.^6^ Better recognition and more accurate description of the course of sepsis is very important for us to improve the prevention, diagnosis, and management of sepsis.

Cardiac troponins regulate muscle contraction which are found in myocardium, composing of three subunits‐Troponin I, T and C.^7^ Cardiac troponin T and I (cTnT and cTnI) have cardiac‐specific isoforms that are only expressed in the adult heart and are used as biomarkers of cardiac injury because of the exquisitely specific pattern of expression.^8^ The assessment of cardiac troponins is not only conducive to the early diagnosis of acute myocardial infarction but also tried for other applications including evaluating of cardiovascular diseases (CVD) risk among healthy people, monitoring myocardial damage at the early stages of CVD and noncardiac conditions, and assisting the diagnosis of CVD via noninvasive methods.^9^ Cardiac dysfunction induced by sepsis is a common clinical challenge and is associated with adverse outcomes and increased mortality rates.^10^ The result of current studies showed that the increasing level of cardiac troponin was a common finding in septic patients.^11^

However, the effect of cardiac troponin on short‐term and long‐term prognosis of patients with sepsis is unknown. Though several studies reported that increased cardiac troponin level was associated with poorer outcomes,^12^, ^13^ these were not confirmed in similar studies.^14^, ^15^ Thus, we conducted this meta‐analysis and analyzed the role of cardiac troponin in patients with sepsis, which may be an indicator for sepsis mortality.

## 2 METHODS

### 2.1 Search strategy

Two researchers (Peiqiu Zheng and Wei Gao) conducted the literature search, considering articles published before November 2022, using Google Scholar, PubMed, and Web of Science. The search terms used were “troponin” AND (“sepsis” OR “septicemia” OR “septic”).

### 2.2 Selection criteria

We established inclusion and exclusion criteria for our study. No language restriction was applied. The inclusion criteria were studies that investigated cardiac troponin and sepsis. On the other hand, the exclusion criteria were applied to remove studies that did not meet our research objectives. These included: (1) studies were excluded when we could not obtain or calculate hazard ratio (HR) and 95% confidence interval (CI) regarding association between cardiac troponin level and mortality of sepsis; (2) reviews, meta‐analysis and case reports. Two researchers, Peiqiu Zheng and Wei Gao, conducted the initial review of all the abstracts and full texts, independently. Subsequently, the three authors, Peiqiu Zheng, Wei Gao, and Qiang Huang, collectively assessed and decided on the included articles after identifying any inconsistent selections.

### 2.3 Data extraction

The data extraction process was performed by two additional researchers, Xing Wang and Tao Guo. The extracted data included the following variables: author, publication year, study design, study location, study population, sample size, and mean age, gender, patients admitted in shock, exclusion of patients with comorbidities, follow‐up time, Cut‐off value of cardiac troponin, adjusted variables and results.

### 2.4 Meta‐analysis

To analyze the association between cardiac troponin levels and mortality, hospital mortality or long‐term mortality in sepsis, we utilized STATA 12.0 software to summarize HR and 95% confidence intervals (CI). For the analysis of the data, a fixed effects model was employed when there was low heterogeneity (indicated by a *p* > .05 for the Q test and *I* ^2^ < 50%). Conversely, a random effects model was utilized when there was high heterogeneity (indicated by a *p* ≤ .05 for the Q test and *I* ^2^ ≥ 50%). Furthermore, we conducted meta‐regression and subgroup analyses to explore the sources of heterogeneity, considering different study types and races. To determine the stability of the studies, a sensitivity analysis was conducted. Additionally, we evaluated publication bias using funnel plots and conducted Begg's and Egger's tests.

## 3 RESULTS

### 3.1 Study characteristics

**Figure 1:**
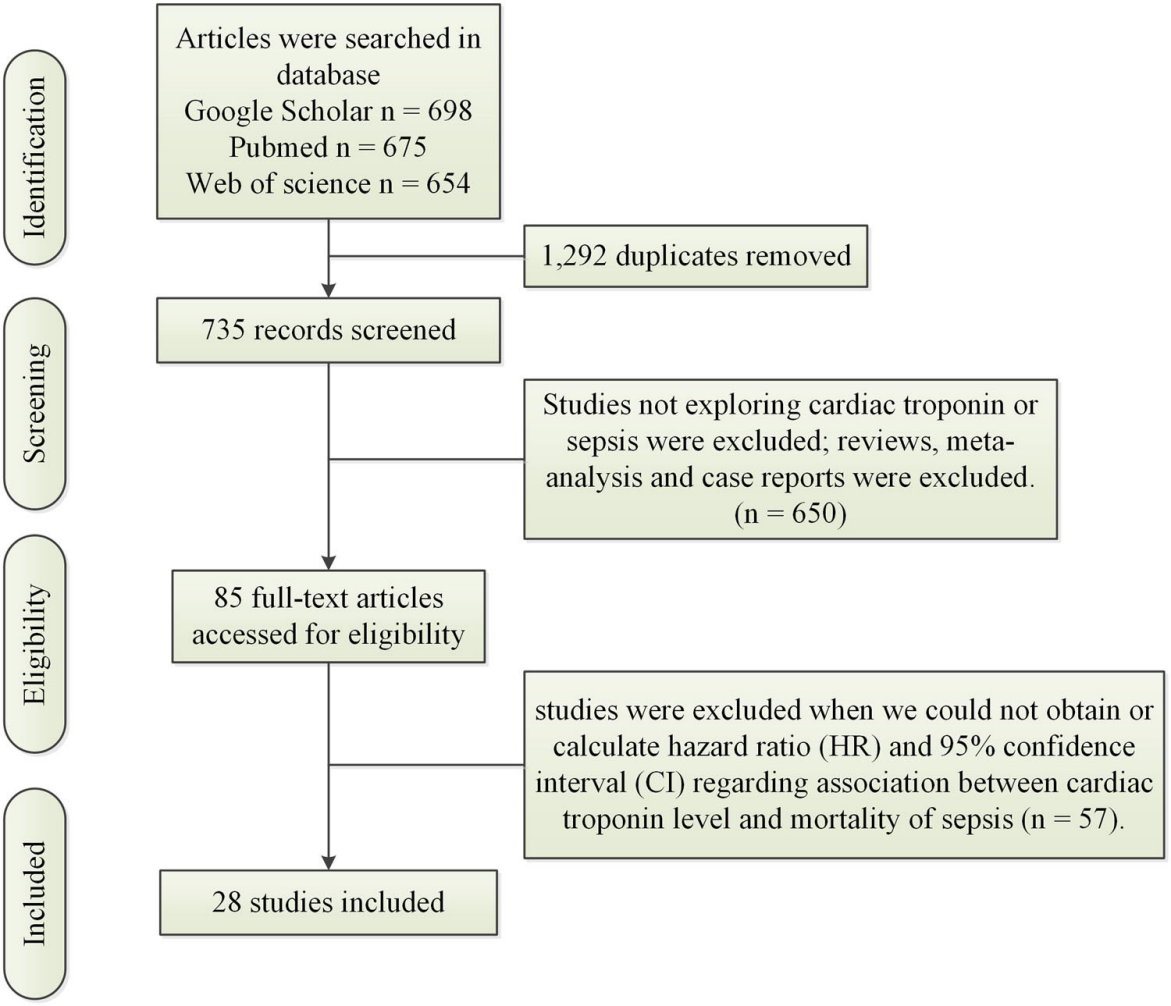
Selection procedures.

Initially, we identified a total of 2027 articles from Google Scholar (*n* = 698), Pubmed (*n* = 675), and Web of Science (*n* = 654). After removing 1292 duplicate articles, we screened the titles and abstracts of 735 articles. Out of these, 614 articles were excluded due to their lack of relevance, leaving us with 121 articles that met the inclusion criteria. We excluded 36 reviews, meta‐analyses, and case reports. Furthermore, 57 articles were excluded because they lacked a control group (15 articles) or provided insufficient data (42 articles). Please refer to Table 1 for study characteristics, while Figure 1 illustrates the selection procedures. Ultimately, our study included a total of 24 prospective studies^12^, ^14^, ^15^, ^16^, ^17^, ^18^, ^19^, ^20^, ^21^, ^22^, ^23^, ^24^, ^25^, ^26^, ^27^, ^28^, ^29^, ^31^, ^33^, ^34^, ^35^, ^36^, ^37^, ^38^ (including *N* = 20,457 sepsis patients) and *N* = 4 retrospective studies^1^, ^30^, ^32^, ^39^ (including *N* = 1416 sepsis patients).

**Table 1:** Characteristics of all included studies.

References	Study design	Study location	Population	Sample Size	Age, SD (years)	Male (%)	Patients admitted in shock (%)	Exclusion of patients with comorbidities	Follow‐up (days)	Cut‐off value of TnI (ng/mL)	Adjustment factor	Results (HR, 95% CI)
Spies et al.^16^	Prospective	ICU, Germany	Sepsis (38% Peritonitis)	26	59.6 (Range 21−89)	58%	NA	Yes	7	0.2	No	3.125 (0.448− 24.661)
Fernandes et al.^17^	Prospective	ICU, Brazil	Sepsis (40% Peritonitis)	10	30.1 ± 5.6	60%	NA	Yes	NR	0.6	No	3 (0.12−209.12)
Turner et al.^18^	Prospective	ICU, Australia	Sepsis (53% Pulmonary)	15	63.8 ± 16.1	NA	100%	Yes	28	NR	No	2.77 (0.19−41.13)
ver Elst et al.^19^	Prospective	ICU, Belgium	Sepsis (74% Pulmonary, 13% Peritonitis)	46	66 (range 54−74)	65%	100%	Yes	NR	NR	No	2.0 (0.99−4.03)
Ammann et al.^20^	Prospective	ICU, Switzerland	Sepsis (55% Pulmonary)	20	66.1 ± 8.2	75%	40%	Yes	Hospital discharge	0.1	No	0.833 (0.035−58.878)
Ammann et al.^21^	Prospective	ICU, Switzerland	Sepsis	58	55 ± 21	48%	41%	Yes	30	0.1	No	4.0 (1.2−8.9)
Mehta et al.^22^	Prospective	ICU, USA	Sepsis	37	68.5 ± 14.2	54%	100%	Yes	Hospital discharge	1	No	5.333 (1.056−28.349)
Brivet et al.^23^	Prospective	ICU, France	Sepsis (60.3% Pulmonary)	136	70.3 ± 15	75%	65.4%	Yes	Hospital discharge	0.3	No	2.56 (1.89−5.542)
John et al.^24^	Prospective	ICU, USA	Sepsis	105	57 ± 15.0	59%	68%	No	28	0.4	No	2.557 (1.066−6.175)
Bouhemad et al.^25^	Prospective	ICU, France	Sepsis (46% Pulmonary)	54	56 ± 17	76%	100%	Yes	10	0.2	No	4.511 (0.849−30.057)
Choon‐ngarm et al.^26^	Prospective	Department of medicine, Thailand	Sepsis (57.5% Pulmonary)	40	60 ± 19.7	NR	100%	Yes	NR	NR	No	2.03 (1.33−3.10)
Issa et al.^27^	Prospective	ICU, Brazil	Sepsis (43.5% Pulmonary)	23	51.3 ± 18.6	60.9%	82%	Yes	ICU discharge	NR	No	1.22 (0.62−2.39)
Scott et al.^28^	Prospective	ICU, USA	Sepsis (53% Abdomen, 20% Pulmonary, 12% Necrotizing fasciitis)	66	64.6 ± 17	59%	64%	No	Hospital discharge	0.1	No	1.52 (0.41−6.37)
Kang et al.^29^	Prospective	Internal medicine (ESRD), Korea	Sepsis (66% Pulmonary, 11.5% Urinary)	121	66.1 ± 11.8	44%	24.8%	Yes	90	0.2	age, diabetes, previous CVD history, presence of septic shock, serum CRP levels and elevated cTnI levels	All‐cause mortality: 5.90 (2.06−16.87); Cardiovascular mortality: 5.17 (1.16–23.16)
John et al.^30^	Retrospective	ICU, PROWESS trial, 11 countries	Sepsis	598	60 ± 17.5	56%	NR	No	28	0.06	Treatment, age, race, functional dependency status, APACHE II, activated partial thromboplastin time, IL‐6, tropo (+)	2.020 (1.153−3.541)
Rosjo et al.^31^	Prospective	ICU, Finland	Sepsis	207	65 (range 56−76)	68%	NR	Yes	Hospital discharge	NR	SAPS II score, tropo (+)	1.29 (1.03−1.60)
Tiruvoipati et al.^32^	Retrospective	ICU, Australia	Sepsis	293	70 (Range 59−78)	50.2%	NR	No	Hospital discharge	0.1	Mean BP, temperature, lactate, SAPS II score	1.61 (0.75−3.46)
Vasile et al.^33^	Prospective	ICU, USA	Sepsis	926	69 (57−78)	56.3%	NR	Yes	Hospital discharge	0.01	No	1.20 (1.12−1.28)
Sasko et al.^15^	Prospective	ICU, Germany	Sepsis	52	71.4 ± 8.5	59.6%	100%	Yes	28	0.015	age, gender, history of coronary artery disease and all hemodynamic variables	1.0 (0.9−1.0)
Masson et al.^34^	Prospective	ICU, 40 centers, Italy	Sepsis	995	65.1 ± 14.3	60%	54.1%	Yes	Hospital discharge, 90	NR	continuous variables (age, body mass index, heart rate, mean arterial pressure, central venous pressure, urine output, lactate, hemoglobin, serum creati‐ nine, platelet count, and central venous oxygen saturation) and categorical variables (sex, reason for admission to ICU, chronic renal failure, immunodeficiency, congestive or ischemic heart disease, shock, treatment assignment, and positive blood culture)	ICU mortality: shock: 3.48 (2−6.06); no shock: 1.90 (1.06−3.4); 90‐day mortality: shock: 2.08 (1.29−3.35); no shock: 1.19 (0.71−2.0);
Yang et al.^35^	Prospective	ICU, USA	Sepsis	375	58	58%	100%	Yes	7	0.05	age	1.9 (1.02–3.83)
Vallabhajosyula et al.^36^	Prospective	ICU, USA	Sepsis	1688	68.14	54%	NR	Yes	Hospital discharge, 365	0.01	Age, sex, body mass index, Charlson comorbidity index, APACHE‐III score, acute kidney injury, and respiratory failure.	Hospital Mortality: 1.4 (1.1−2.1); 1‐year Mortality: 1.3 (1.1−1.6)
Frencken et al.^37^	Prospective	ICU, The Netherlands	Sepsis	1124	62.5	62%	25.5%	Yes	ICU, Hospital discharge, 365	0.026	No	ICU Mortality: 2.163 (1.529−3.087); Hospital Mortality: 1.945 (1.468− 2.585); 1‐year Mortality: 1.757 (1.367− 2.259)
Abdalla et al.^14^	Prospective	ICU, USA	Sepsis (80% Respiratory)	125	72	63.3%	50%	Yes	Hospital discharge	NR	NR	1.6 (0.78−3.4)
Garcia et al.^38^	Prospective	ICU, USA	Sepsis	14,046	74	48%	NR	Yes	Hospital discharge	0.04	age, body mass index, sex, race, Charlson comorbidity score and history of diabetes mellitus, chronic kidney disease, dialysis, proteinuria, renal disease, chronic pulmonary disease, et al.	Abnormal tertile 1: 1.04 (0.94−1.15); Abnormal tertile 2: 1.08 (0.97−1.19); Abnormal tertile 3: 1.06 (0.95−1.18)
Mu et al.^39^	Retrospective	ICU, China	Sepsis	300	NR	NR	NR	Yes	28	NR	NR	2.115 (1.189‐ 5.459)
Forner et al.^12^	Prospective	ICU, Germany	Sepsis	162	70 (61−78)	65.4%	42.6%	Yes	30	0.14	NR	1.703 (1.030–2.814)
Wen et al.^1^	Retrospective	ICU, China	Sepsis	225	67 (52−78)	61.3%	32.9%	Yes	28	0.1	No	5.50 (2.615–11.536)

Abbreviations: BP, blood pressure; CI, confidence interval; CRP, C‐reactive protein; cTnI, Cardiac troponin I; CVD, cardiovascular disease; ESRD, end‐stage renal disease; HR, hazard ratio; ICU, intensive care unit; IL‐6, interleukin‐6; NA, not applicable; NR, not reported; PROWESS, Protein C Worldwide Evaluation in Severe Sepsis; SAPS, simplified acute physiology score; SD, standard deviation; USA, United States.

### 3.2 Cardiac troponin level and mortality of sepsis

**Figure 2:**
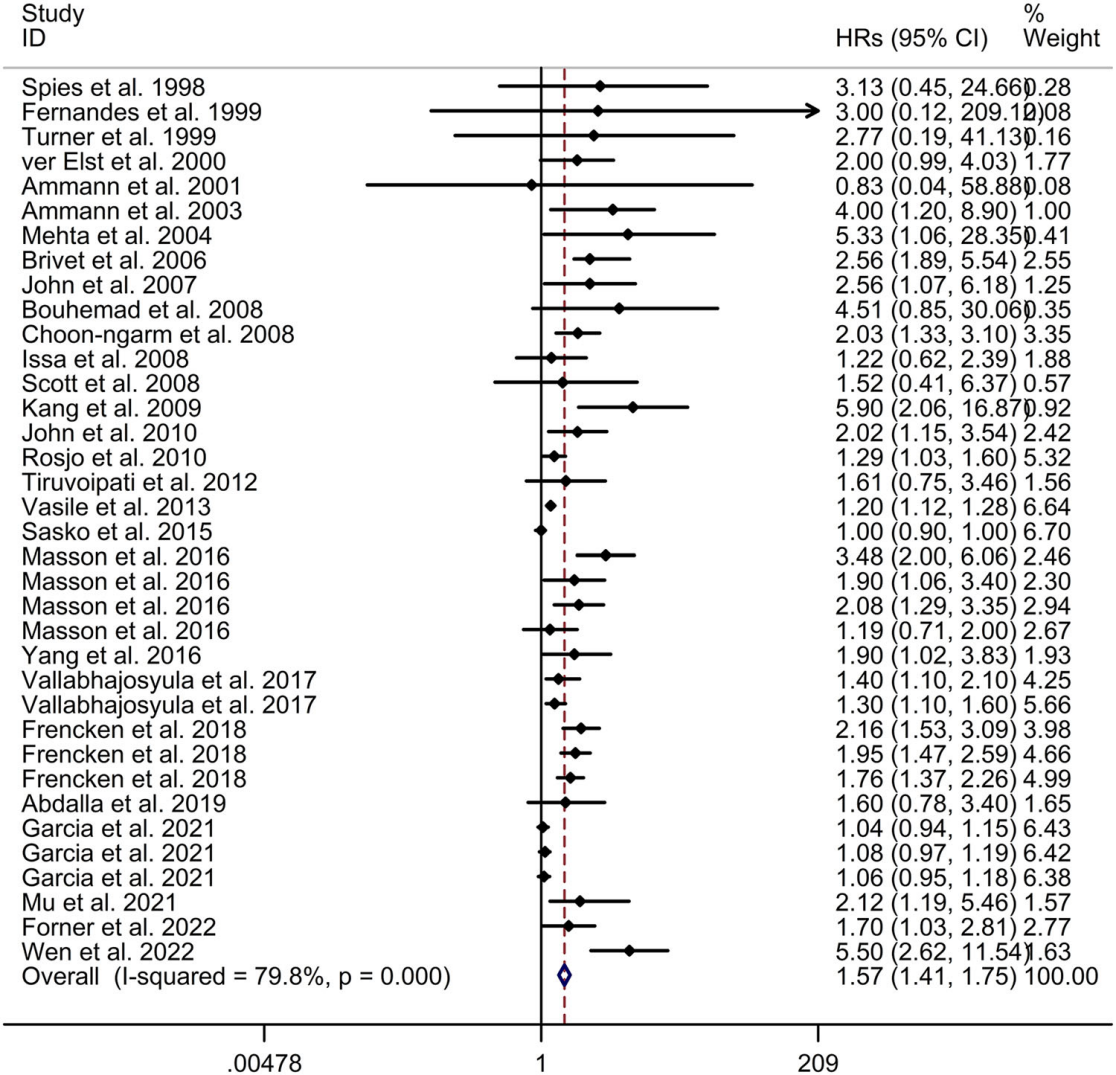
Forest plot regarding association between cardiac troponin level and mortality of sepsis. CI, confidence interval; HR, hazard ratio.

**Figure 3:**
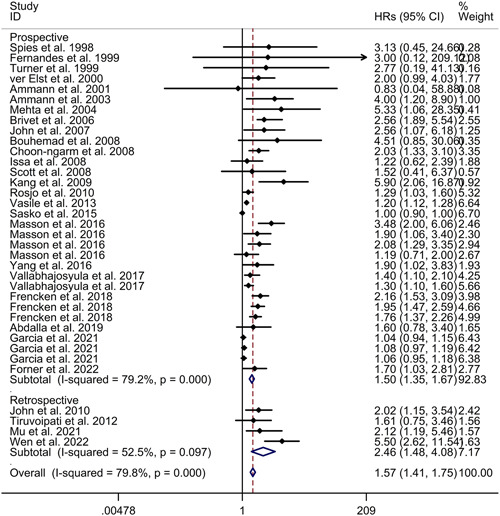
Subgroup analysis regarding association between cardiac troponin level and mortality of sepsis in different types of studies. CI, confidence interval; HR, hazard ratio.

**Figure 4:**
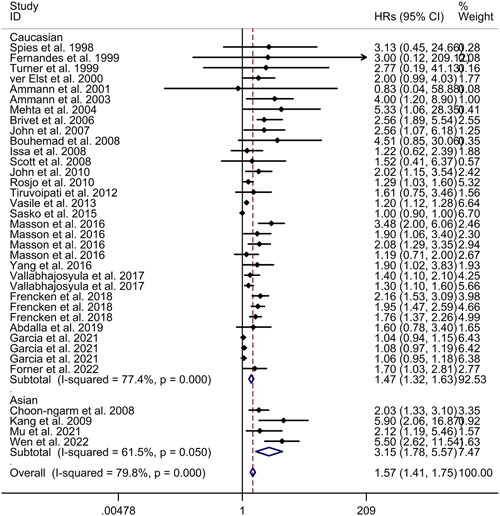
Subgroup analysis regarding association between cardiac troponin level and mortality of sepsis from different races. CI, confidence interval; HR, hazard ratio.

Our meta‐analysis revealed that there is an association between cardiac troponin elevation and increased mortality in sepsis, as indicated by a random effects model (HR = 1.57, 95% CI 1.41−1.75, *I* ^2^ = 79.8%, *p* < .001; see Figure 2). However, the meta‐regression analysis demonstrated that age, gender, and patients admitted in shock were not responsible for the high heterogeneity observed between the included studies (age: *p* = .802; gender: *p* = .497; patients admitted in shock: *p* = .173). Subgroup analysis results indicated that cardiac troponin elevation was associated with increased mortality in sepsis, both in prospective studies (HR = 1.50, 95% CI 1.35−1.67) and retrospective studies (HR = 2.46, 95% CI 1.48−4.08; see Figure 3). Furthermore, subgroup analysis revealed consistent results for both Caucasian populations (HR = 1.47, 95% CI 1.32−1.63) and Asian populations (HR = 3.15, 95% CI 1.78−5.57; see Figure 4). Sensitivity analysis indicated that the exclusion of any single study did not alter the direction of effect (see Supporting Inforrmation: Figure 1). The funnel plot, Begg's test, and Egger's test all indicated a significant risk of publication bias (see Supporting Inforrmation: Figure 2; Begg's test: *p* < .001; Egger's test: *p* < .001).

### 3.3 Cardiac troponin level and hospital mortality of sepsis

**Figure 5:**
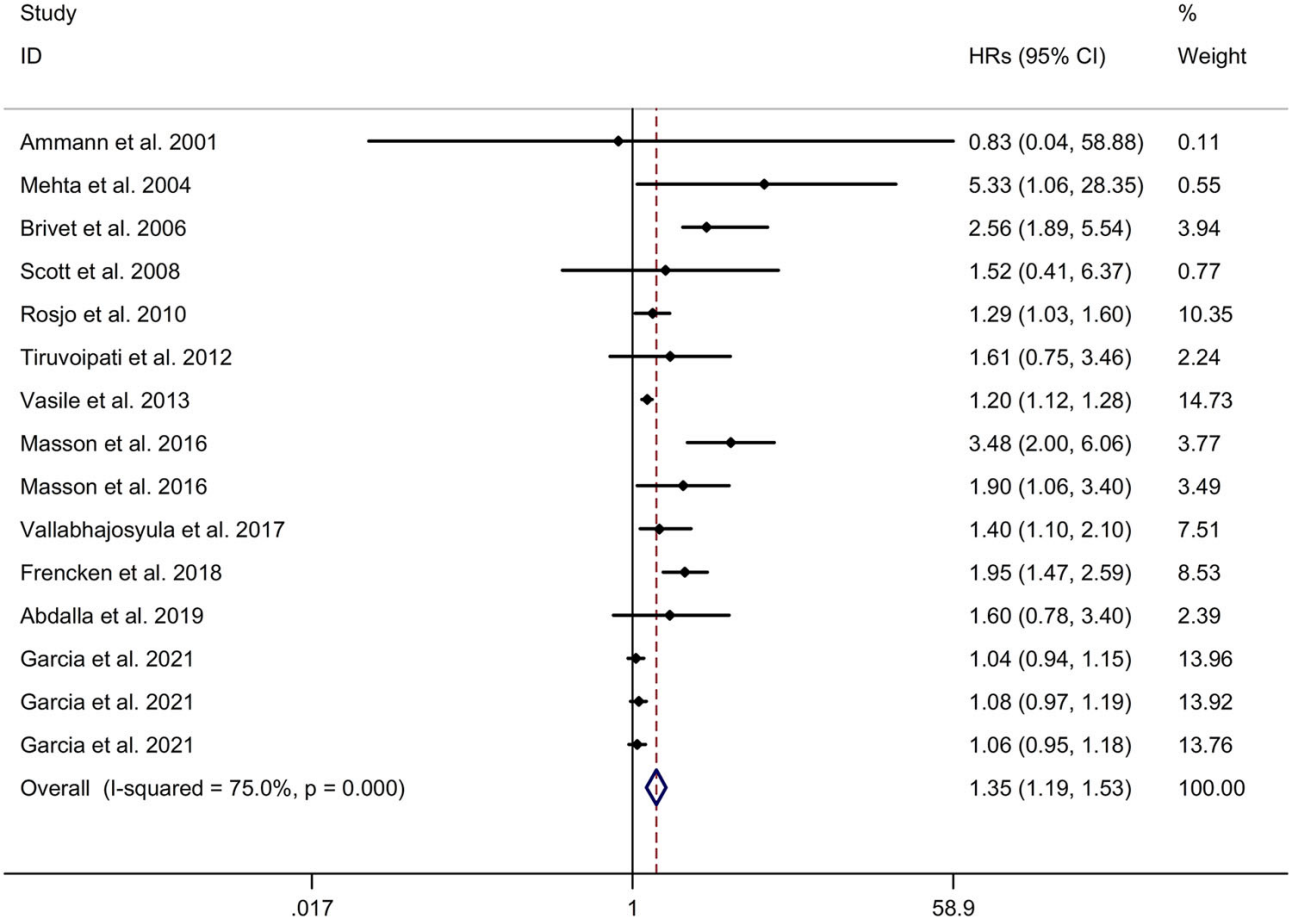
Forest plot regarding association between cardiac troponin level and hospital mortality of sepsis. CI, confidence interval; HR, hazard ratio.

The association between cardiac troponin level and hospital mortality of sepsis was investigated through a meta‐analysis, which revealed that cardiac troponin elevation was significantly linked to an increased hospital mortality of sepsis. The analysis used a random effects model with a HR of 1.35 and a 95% CI of 1.19−1.53 (see Figure 5). The heterogeneity among included studies was found to be high, with an *I* ^2^ value of 75.0%. Age, gender, and patients admitted in shock were considered as potential factors contributing to the heterogeneity, but the meta‐regression study showed no significant association between these variables and the observed heterogeneity. Additionally, a subgroup analysis was conducted to explore the association between cardiac troponin elevation and hospital mortality of sepsis in prospective studies, which supported the initial findings (HR = 1.35, 95% CI 1.19−1.53) (see Supporting Information: Figure 3). It is important to note that all sepsis patients included in the study were from the Caucasian population. To evaluate the robustness of the results, a sensitivity analysis was performed by excluding individual studies from the meta‐analysis. The sensitivity analysis did not alter the direction of the effect, further supporting its robustness and reliability, indicating that the overall findings were not influenced by any specific study (see Supporting Information: Figure 4). Furthermore, the presence of publication bias was assessed using a funnel plot, Begg's test, and Egger's test. The results indicated a significant risk of publication bias, suggesting that studies with positive results may have been more likely to be published (see Supporting Information: Figure 5; Begg's test: *p* = .692; Egger's test: *p* = .01).

### 3.4 Cardiac troponin level and long‐term mortality of sepsis

**Figure 6:**
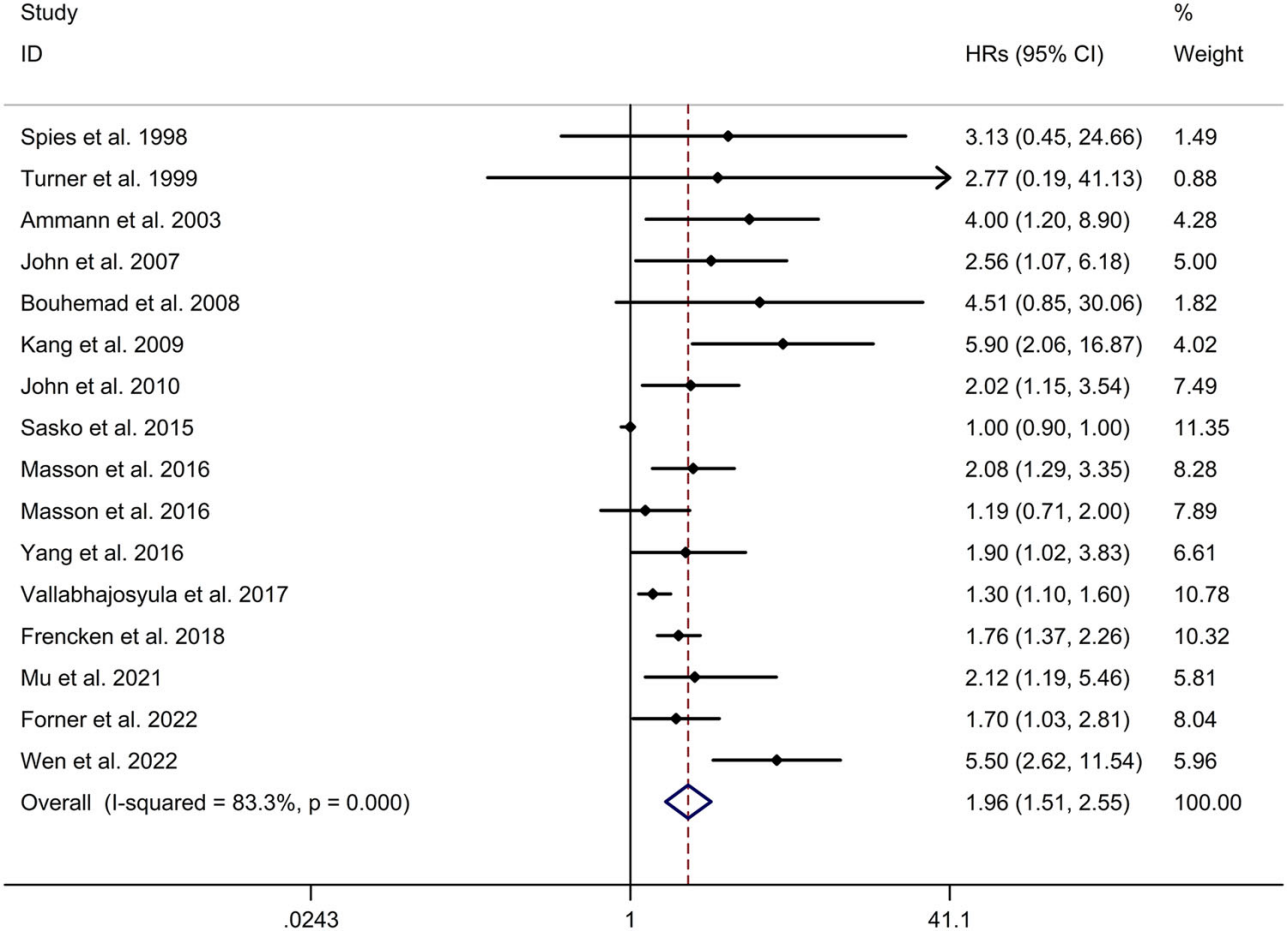
Forest plot regarding association between cardiac troponin level and long‐term mortality of sepsis. CI, confidence interval; HR, hazard ratio.

In the context of long‐term mortality of sepsis, a similar meta‐analysis was conducted, which revealed a significant association between cardiac troponin elevation and increased long‐term mortality. The random effects model showed a HR of 1.96 and a 95% CI of 1.51−2.55 (see Figure 6). The heterogeneity among the included studies was high, with an *I* ^2^ value of 83.3%. Age, gender, patients admitted in shock, and follow‐up time were analyzed as potential sources of heterogeneity, but the meta‐regression study found no significant associations. Subgroup analyses were performed to explore the association between cardiac troponin elevation and long‐term mortality of sepsis in both prospective and retrospective studies. The results showed that cardiac troponin elevation was linked to increased long‐term mortality in both types of studies, with HRs of 1.76 (95% CI 1.35−2.29) for prospective studies and 2.80 (95% CI 1.50−5.21) for retrospective studies (see Supporting Information: Figure 6). Moreover, the association was observed in both Caucasian and Asian populations, with HRs of 1.67 (95% CI 1.31−2.14) and 3.95 (95% CI 2.01−7.75), respectively (see Supporting Information: Figure 7). Similar to the previous analysis, a sensitivity analysis was conducted to assess the robustness of the results, and no change in the direction of the effect was observed when individual studies were excluded (see Supporting Information: Figure 8). Lastly, the presence of publication bias was evaluated using a funnel plot, Begg's test, and Egger's test. The results indicated a significant risk of publication bias, suggesting that studies with positive results may have been more likely to be published (see Supporting Information: Figure 9; Begg's test: *p* = .558; Egger's test: *p* < .001).

## 4 DISCUSSION

In this meta‐analysis, we all included 28 studies with a total of over 21000 participants (24 prospective and four retrospective studies). Our findings showed that there was a significantly association between cardiac troponin elevation and mortality among septic patients (HR = 1.57, 95% CI 1.41−1.75). We also revealed that the hospital and long‐term mortalities were higher in patients with sepsis (hospital: HR = 1.35, 95% CI 1.19−1.53; long‐term: HR = 1.96, 95% CI 1.51−2.55). All these results indicated that cardiac troponin may act as a potential biomarker of septic patients' outcomes. It is worth mentioning that a previous meta‐analysis conducted by Bessière et al.^40^ also reported a significant association between elevated cardiac troponin and all‐cause mortality in sepsis patients. This meta‐analysis included 13 articles with 1227 patients and found a relative risk (RR) of 1.91 (95% CI 1.63−2.24). In Bessière's paper, 12 articles^16^, ^17^, ^18^, ^19^, ^20^, ^22^, ^23^, ^24^, ^26^, ^27^, ^29^, ^30^ were utilized, which shared similarities with our manuscript. However, Yucel's^41^ was excluded from our analysis due to inadequate data provided for obtaining or calculating the HR and 95% CI concerning the association between cardiac troponin levels and sepsis mortality. In congruence with our findings, Sheyin et al. suggested that sepsis patients with elevated cardiac troponin levels had approximately double the risk of mortality compared to those without such elevation (RR = 1.91, 95% CI 1.65−2.22).^42^ All studies^16^, ^17^, ^18^, ^19^, ^20^, ^21^, ^22^, ^23^, ^24^, ^25^, ^26^, ^27^, ^28^, ^29^, ^30^, ^31^, ^32^ cited in Sheyin's paper also resembled our manuscript, supporting the consistency of previous results with our findings. Furthermore, our study presents updated information on both hospital mortality and long‐term mortality in sepsis patients.

The underlying mechanism of elevated cardiac troponin associated with worsen prognosis of sepsis is unclear up to now. Sepsis induced cardiomyopathy, or sepsis‐induced myocardial dysfunction has become a focus of many studies.^43^ Furthermore, previous in vivo and in vitro studies using sepsis models have demonstrated that sepsis damages the structure and function of mitochondria, leading to the overproduction of mitochondria‐derived danger‐associated molecular patterns (DAMPs) such as reactive oxygen species, fragmented mitochondrial DNA, N‐formyl peptides, cardiolipin, adenosine triphosphate, mitochondrial transcription factor A, and cytochrome c.^44^ These harmful DAMPs contribute to myocardial inflammation, exacerbating the effects of sepsis. Although myocardial ischaemia is not evident as a whole, the microcirculatory endothelial cells in response to inflammation could shut the cardiac microcirculatory flow in hypoperfused microregions, resulting in the cardiac functional abnormalities among sepsis patients.^13^ Vasques‐Nóvoa et al. showed that the association between myocardial edema and tissue injury may explain the changes of sepsis‐induced myocardial injury in part via clinical and histological evidence.^45^

However, there are some limitations in this meta‐study. First, the publication bias indicated the existence of unpublished studies of smaller sample size or negative results, thus searching literature more carefully and contacting authors of unpublished articles are essential for us. Second, most of participants included in this meta‐analysis was middle age and elderly people, we cannot exclude the effect of common metabolic diseases. In addition, it is doubtful whether the finding can apply to young people with sepsis even to patients with neonatal sepsis.

## 5 CONCLUSIONS

Overall, our finding revealed that elevated cardiac troponin for sepsis patients was a predictor of hospital and long‐term mortality. Clinicians may treat septic patients with elevated cardiac troponin more cautious to avoid extra death. Moreover, large clinical studies are warranted to validate this association.

## AUTHOR CONTRIBUTIONS

**Peiqiu Zheng**: Conceptualization; data curation; investigation; methodology; software; writing—original draft. **Xing Wang**: Project administration; resources; software. **Tao Guo**: Formal analysis; investigation; methodology; software. **Wei Gao**: Conceptualization; data curation; software. **Qiang Huang**: Data curation; investigation; methodology; resources; software. **Jie Yang**: Formal analysis; methodology; software; visualization. **Hui Gao**: Formal analysis; methodology. **Qian Liu**: Funding acquisition; supervision; writing—original draft; writing—review and editing.

## CONFLICT OF INTEREST STATEMENT

The authors declare no conflict of interest.

## Supporting information

Supporting information.

Supporting information.

Supporting information.

Supporting information.

Supporting information.

Supporting information.

Supporting information.

Supporting information.

Supporting information.

## Data Availability

The data could be obtained from corresponding author.
