# Supplementary material for: Cardiac troponin as a prognosticator of mortality in patients with sepsis: A systematic review and meta‐analysis
Source: Immun Inflamm Dis. 2023 Sep 22;11(9):e1014. doi: 10.1002/iid3.1014 (PMC10515504; doi:10.1002/iid3.1014)
Supplement: Supplementary file 2 — Supporting information. [file IID3-11-e1014-s002.docx]

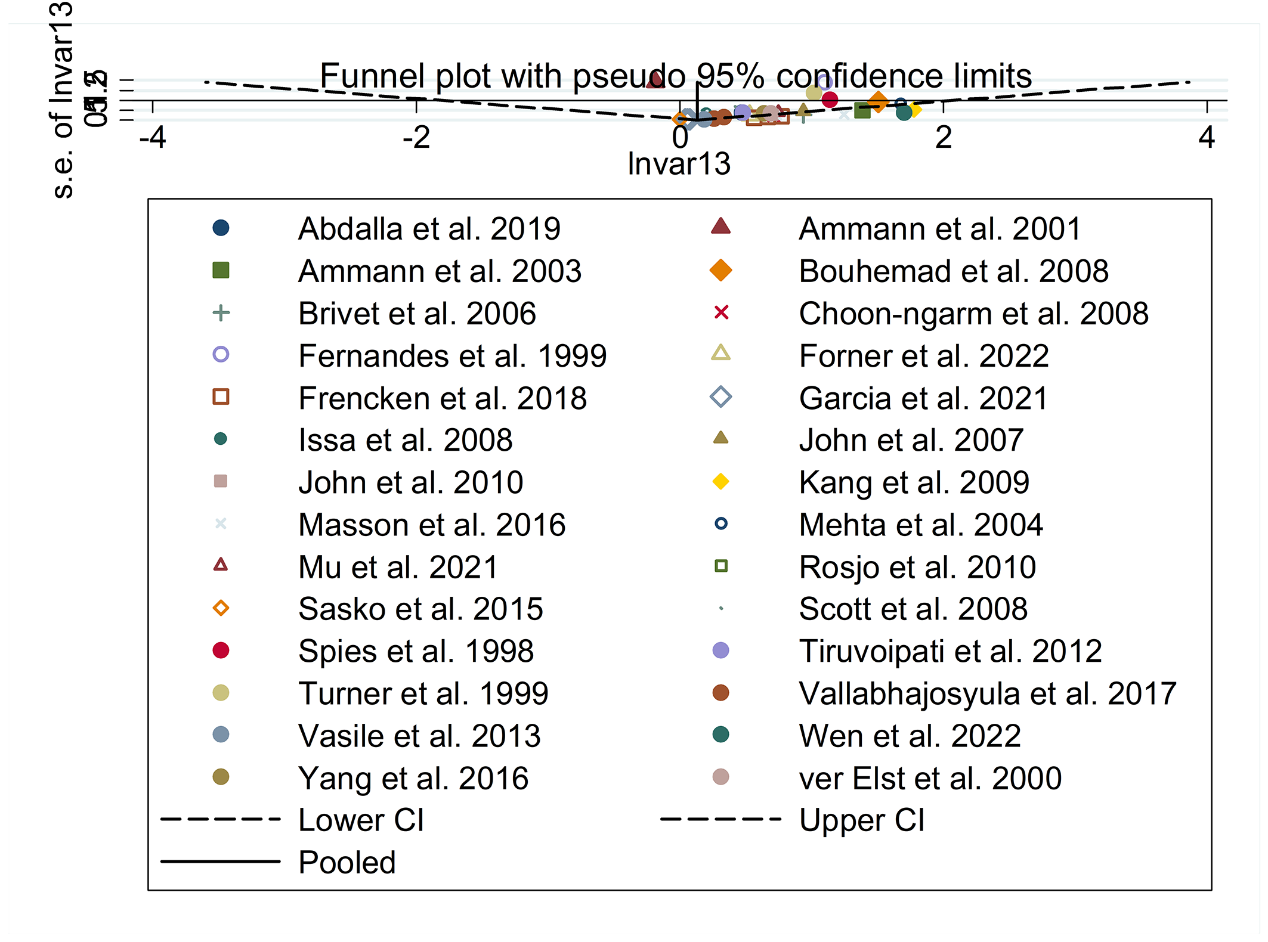


Supplementary figure 2. Funnel plot regarding association between cardiac troponin level and mortality of sepsis.
